# Transient Hemodynamic Changes upon Changing a BCPA into a TCPC in Staged Fontan Operation: A Computational Model Study

**DOI:** 10.1155/2013/486815

**Published:** 2013-11-10

**Authors:** Fuyou Liang, Hideaki Senzaki, Zhaofang Yin, Yuqi Fan, Koichi Sughimoto, Hao Liu

**Affiliations:** ^1^SJTU-CU International Cooperative Research Center, School of Naval Architecture, Ocean and Civil Engineering, Shanghai Jiao Tong University, 800 Dongchuan Road, Shanghai 200240, China; ^2^Department of Pediatrics and Pediatric Cardiology, Saitama Medical Center, Saitama Medical University, Staff Office Building 101, 1981 Kamoda, Kawagoe, Saitama 3508550, Japan; ^3^Department of Cardiology, Shanghai Ninth People's Hospital, Shanghai Jiao Tong University School of Medicine, 639 Zhizaoju Road, Shanghai 200011, China; ^4^Department of Cardiac Surgery, Royal Children's Hospital, 50 Flemington Road, Parkville Victoria, Melbourne, VIC 3052, Australia; ^5^Graduate School of Engineering, Chiba University, 1-33 Inage, Chiba 2638522, Japan

## Abstract

The clinical benefits of the Fontan operation in treating single-ventricle defects have been well documented. However, perioperative mortality or morbidity remains a critical problem. The purpose of the present study was to identify the cardiovascular factors that dominate the transient hemodynamic changes upon the change of a bidirectional cavopulmonary (Glenn) anastomosis (BCPA) into a total cavopulmonary connection (TCPC). For this purpose, two computational models were constructed to represent, respectively, a single-ventricle circulation with a BCPA and that with a TCPC. A series of model-based simulations were carried out to quantify the perioperative hemodynamic changes under various cardiovascular conditions. Obtained results indicated that the presence of a low pulmonary vascular resistance and/or a low lower-body vascular resistance is beneficial to the increase in transpulmonary flow upon the BCPA to TCPC change. Moreover, it was found that ventricular diastolic dysfunction and mitral valve regurgitation, despite being well-known risk factors for poor postoperative outcomes, do not cause a considerable perioperative reduction in transpulmonary flow. The findings may help physicians to assess the perioperative risk of the TCPC surgery based on preoperative measurement of cardiovascular function.

## 1. Introduction

The Fontan operation is a surgical procedure performed in patients with complex congenital heart disease that cannot be treated by a biventricular repair [1]. Since the first proposal in 1971 [2], the operation has been undergoing modifications, such as introduction of staged operation strategy, refinement in pre- and post-Fontan management, and optimization of cavopulmonary connection configuration [1]. These modifications have contributed significantly to a continuous improvement of clinical outcomes in the past decades. For example, the long-term (>10 years) survival rate has been increased from 60–70% (before 1985) to 80–90% [3–5]. Moreover, the technical advancements have enabled the extension of the operation to high-risk patients [6].

Despite the overall favorable clinical manifestations, the operation remains to be further improved, particularly in the perioperative stage. A follow-up study revealed that perioperative mortality accounted for 68.4% of all deaths [5]. Another study showed that the rate of early death in high-risk patients reached over 10%, due primarily to low cardiac output syndrome [6]. Population-based studies have allowed identification of many factors associated with poor clinical outcomes, such as low ventricular ejection fraction [7], high pulmonary vascular resistance [8, 9], atrioventricular valve regurgitation [6, 8], and ventricular diastolic dysfunction [10, 11]. The hemodynamic effect of a specific factor is, however, difficult to be quantified by clinical measurements because under in vivo conditions multiple factors always interact to determine the overall hemodynamic conditions in the cardiovascular system. At this point, computational modeling of the cardiovascular system may offer a useful complementary tool for clinical studies. In the literature, computational models have been widely used to provide quantitative insights into hemodynamic phenomena of interest [12–14], including those related to the Fontan operation [15–18].

In the present study, we developed a set of computational models to account for the hemodynamic characteristics of a single-ventricle circulation in different stages of Fontan operation: (1) in the second stage with a bidirectional cavopulmonary (Glenn) anastomosis (BCPA) and (2) in the final stage with a total cavopulmonary connection (TCPC). The models were used to quantify the transient changes in hemodynamic variables, such as central venous pressure and oxygenated flow (i.e., flow through the pulmonary circulation) upon the change of a BCPA into a TCPC. In particular, we carried out a series of simulations under various cardiovascular conditions, with the aim of identifying the cardiovascular factors that dominate perioperative hemodynamic changes.

## 2. Materials and Methods

### 2.1. Model Development

**Figure 1:**
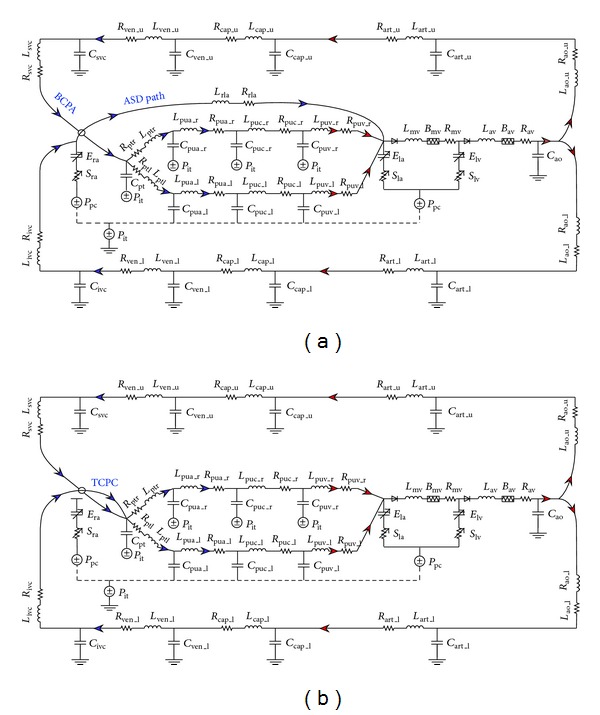
Electric analogies of the single-ventricle circulations with a BCPA (a) and a TCPC (b). The arrows indicate the direction of blood flow.

A single-ventricular circulation with a BCPA was firstly modeled using the lumped parameter modeling method. In the model, the pulmonary circulation was divided into the left and right parts, with each part being further divided into three serially arranged compartments that represent the arterial, capillary, and venous vascular portions, respectively (see Figure 1(a)). The systemic vascular system was divided into aorta, vena cava, and two parallel-arranged upper-body (including the head and the upper limbs) and lower-body (including the splanchnic organs and the lower limbs) subsystems. The properties of each vascular portion were accounted for by three parameters (namely, resistance (*R*), compliance (*C*), and inertance (*L*)) that represent the viscous resistance, wall deformability, and blood inertia of the vascular portion, respectively. Governing equations were obtained by imposing mass and momentum conservation along the flow pathway. Taking blood flows in the vicinity of the lower-body capillary bed as an example, the mass conservation equation reads

(1) $$\begin{array}{c} \frac{dP_{\text{cap}\_\text{l}}}{dt}=\frac{Q_{\text{art}\_\text{l}}-Q_{\text{cap}\_\text{l}}}{C_{\text{cap}\_\text{l}}}, \end{array}$$

and the momentum conservation equation is

(2) $$\begin{array}{c} \frac{dQ_{\text{cap}\_\text{l}}}{dt}=\frac{P_{\text{cap}\_\text{l}}-Q_{\text{cap}\_\text{l}}R_{\text{cap}\_\text{l}}-P_{\text{ven}\_\text{l}}}{L_{\text{cap}\_\text{l}}}. \end{array}$$

Here, *P*_cap_l_ and *P*_ven_l_ refer, respectively, to the blood pressure in the lower-body capillary bed and the veins; *C*_cap_l_, *R*_cap_l_, and *L*_cap_l_ represent, respectively, the compliance, viscous resistance, and blood inertance of the lower-body capillary bed; *Q*_cap_l_ and *Q*_art_l_ denote the blood flow rates through the lower-body capillary bed and the upstream arteriolar bed, respectively.

According to the typical anatomy of a single-ventricle heart, the heart was herein modeled to include three chambers, namely, the right atrium, the left atrium, and the left ventricle (the left ventricle is herein taken as the functional ventricle). Moreover, there is an atrial septal defect (ASD) located between the right and left atria. The pumping action of each cardiac chamber was described by a time-varying elastance that has been widely used in previous studies [19–22]:

(3) $$\begin{array}{c} E\left(t\right)=E_{s}e\left(t\right)+E_{d}, \end{array}$$

where *E*_*s*_ is the maximum value of the active elastance; *E*_*d*_ is the baseline stiffness of the cardiac chamber. For the left ventricle, *E*_*s*_ and *E*_*d*_ reflect the systolic and diastolic functions, respectively. *e*(*t*) is a normalized time-varying function of the active elastance; for the left ventricle, it is written as [22]

(4) elv(t)={0.5[1−cos⁡⁡(πtTvcp)],0≤t≤Tvcp,0.5{1+cos⁡⁡[π(t−Tvcp)Tvrp]},Tvcp<t≤Tvcp+Tvrp,0,Tvcp+Tvrp<t≤T0.

**Figure 2:**
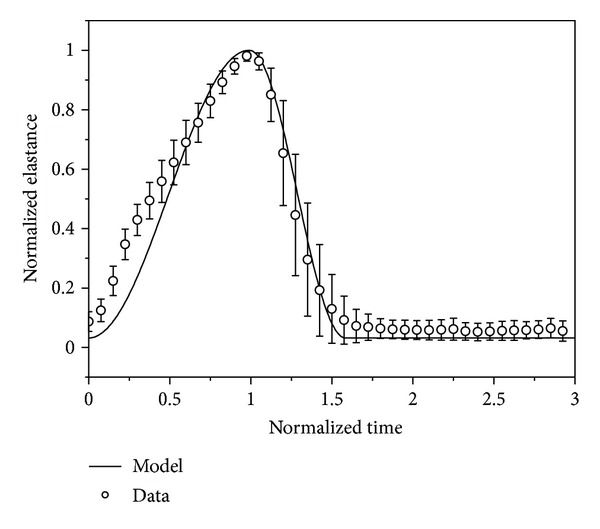
Modeled elastance curve (*E*(*t*)) of the left ventricle compared with in vivo data (mean ± SD) under resting conditions (heart rate = 75 beats/min). Here, the elastance is normalized by its peak value and time is normalized by the time interval from the beginning of ventricular contraction to the arrival of the peak elastance.

Here, *T*_0_ is the duration of a cardiac cycle; *T*_vcp_ and *T*_vrp_ refer, respectively, to the durations of ventricular contraction and relaxation. The modeled elastance curve of the left ventricle under resting conditions (heart rate = 75 beats/min) is compared with in vivo data [23] in Figure 2.

With the elastance being defined, blood pressure (*P*_cc_) in each cardiac chamber can be related to chamber volume (*V*_cc_) by [19–22]

(5) $$\begin{array}{c} P_{\text{cc}}\left(t\right)=E\left(t\right)\left(V_{\text{cc}}-V_{\text{cc},0}\right)+S_{\text{cc}}\frac{dV_{\text{cc}}}{dt}, \end{array}$$

where *V*_cc,0_ refers to the unstressed volume, herein taken to be zero, and *S*_cc_ is the viscoelasticity coefficient of the cardiac wall. 

The hemodynamic effects of cardiac valves were modeled by means of relating the pressure gradient across the valves to the transvalve flow rates [19, 22]. Taking the mitral valve as an example,

(6) $$\begin{array}{c} \Delta P_{\text{mv}}=R_{\text{mv}}Q_{\text{mv}}+B_{\text{mv}}Q_{\text{mv}}\left|Q_{\text{mv}}\right|+L_{\text{mv}}\frac{dQ_{\text{mv}}}{dt}, \end{array}$$

where Δ*P*_mv_ and *Q*_mv_ represent the transvalve pressure drop and flow rate, respectively. *R*_mv_, *B*_mv_, and *L*_mv_ refer, respectively, to the transvalve viscous resistance, Bernoulli's resistance, and blood inertance when the mitral valve is opened.

A normal mitral valve will close when atrial pressure goes below the ventricular pressure and, at the same time, transmitral flow approaches zero, thus effectively preventing the occurrence of reversed flow directed from the left ventricle toward the left atrium. However, such a role may be weakened when pathological changes develop in the leaflets of the mitral valve. A typical phenomenon associated with mitral valve abnormalities is flow regurgitation in systole. To model the phenomenon, we calculated the mitral valve Bernoulli's resistance (*B*_mv,reg_) and inertance (*L*_mv,reg_) upon the occurrence of flow regurgitation based on the effective area of flow regurgitation (*A*_reg_) and the dimension of the left atrium:

(7) $$\begin{array}{c} B_{\text{mv},\text{reg}}=0.5\rho {\left(\frac{1}{A_{\text{reg}}}-\frac{1}{A_{\text{la}}}\right)}^{2},\\ L_{\text{mv},\text{reg}}=2\pi \rho \sqrt{\frac{1}{A_{\text{reg}}}-\frac{1}{A_{\text{la}}}}, \end{array}$$

where *ρ* is the density of blood; *A*_la_ is the nominal area of the left atrium calculated from its volume by assuming a spherical shape.

In this way, a mitral valve with regurgitation operates in two modes: (1) the normal mode where the transvalve forward flow is computed using the normal valve parameters and (2) the regurgitation mode in which case the atrium to ventricle retrograde flow is computed using the valve parameters derived from (7).

The governing equations consisted mainly of the ordinary differential equations formulated at the *R*, *L*, and *C* components and nonlinear equations that describe cardiac chamber dynamics and cardiac valve function. The equation system was solved using a fourth-order Runge-Kutta method. For more details, the reader is invited to refer to our previous studies [20, 21].

### 2.2. Parameter Assignment

The parameters used in the models were assigned to reproduce the typical hemodynamic characteristics of a child aged 3-4 years with the weight and height being 17 Kg and 105 cm, respectively. The total vascular compliance (*C*_sys_) and pulmonary vascular compliance (*C*_pul_) were set as a function of body weight by *C*_sys_ = 2.1 ∗ weight [24] and *C*_pul_ = 0.408 ∗ weight, respectively [25]. For other model parameters, such as systemic/pulmonary vascular resistances and cardiac elastances, although most of them are derivable from previous studies [26–28], there are significant discrepancies among the values used in different studies. Therefore, the model parameters were reassessed in the present study. To this aim, preliminary parameter estimation from a validated adult model [20, 21] was firstly performed based on the weight and body surface area according to the general scaling laws [29]. Subsequently, the parameter values were refined via a parameter optimization procedure [30] aimed to match model-simulated hemodynamic variables with the available in vivo data [18, 31–33]. The assigned parameter values are summarized in Table 1, which were used both in the BCPA circulation model and in the TCPC circulation model. The simulated results for the BCPA circulation under resting conditions (cardiac duration = 0.67 s) are compared with clinical data in Table 2. It is observed that all the model-simulated hemodynamic values fall within the ranges of the measured data.

### 2.3. Simulation Conditions

Model parameters corresponding to the major risk factors identified in clinical studies were studied regarding their effects on the hemodynamic consequence of changing a BCPA into a TCPC. The parameters studied include the pulmonary vascular resistance (*R*_pul_), the arteriolar resistance of the lower body (*R*_art_l_), and the ventricular maximum active elastance (*E*_slv_) in systole and baseline passive elastance (*E*_dlv_) in diastole. In each set of simulation, a parameter varied from 50% to 250% of its default value in an interval of 10%. It is noted that, when a parameter varied, the other three parameters were held at their default values (as given in Table 1). Moreover, the effects of mitral valve regurgitation were investigated as well by varying the effective area of flow regurgitation from 0.01 to 0.21 cm^2^. In all the simulations, the cardiac duration (*T*_0_), intrathoracic pressure (*P*_it_), and pericardial pressure (*P*_pc_) were fixed at 0.67 s, −3.5 mmHg, and 3 mmHg, respectively.

## 3. Results

### 3.1. Hemodynamic Changes Associated with the Change of a BCPA to a TCPC

**Figure 3:**
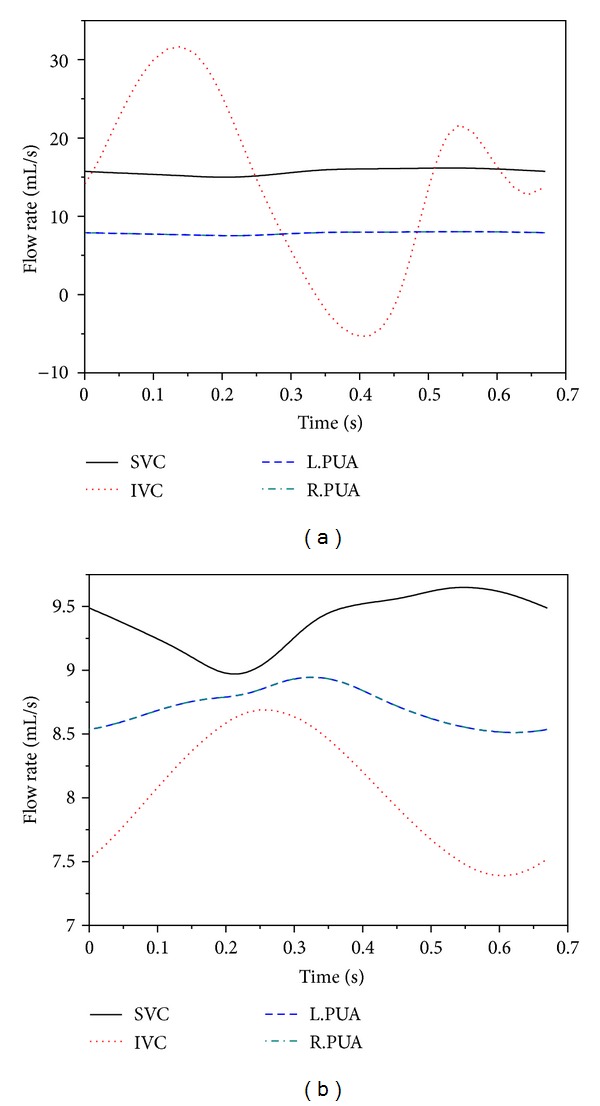
Simulated flow waveforms in the vena cava and pulmonary arteries for the BCPA circulation (a) and TCPC circulation (b). SVC: superior vena cava; IVC: inferior vena cava; L.PUA: left pulmonary artery; R.PUA: right pulmonary artery.

**Figure 4:**
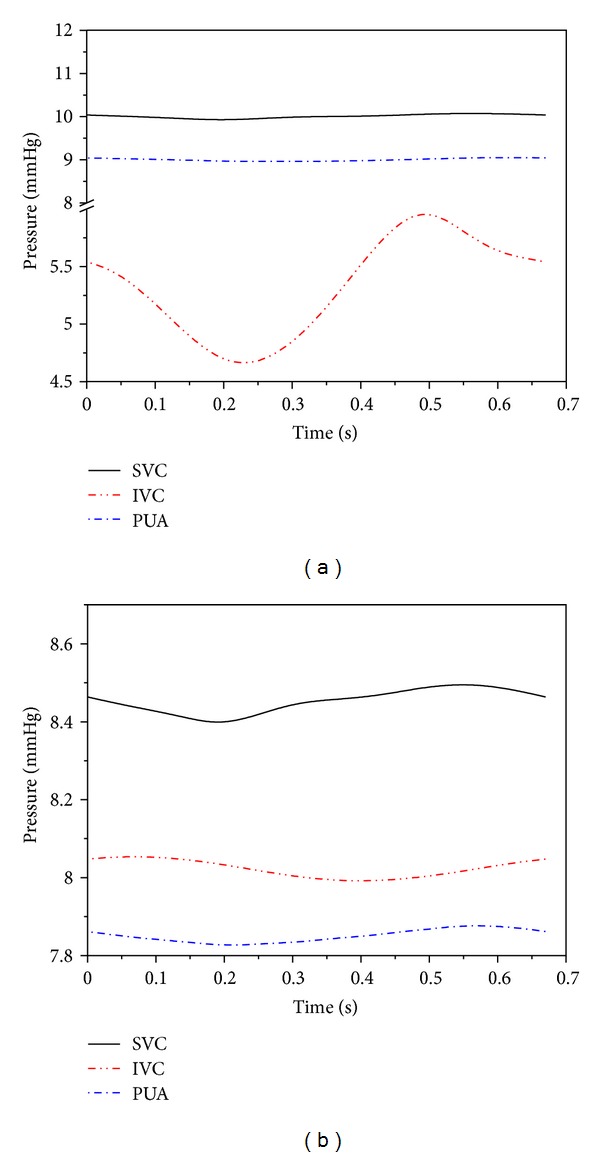
Simulated pressure waveforms in the vena cava and pulmonary arteries for the BCPA circulation (a) and TCPC circulation (b).

Simulations were firstly performed for the BCPA circulation and the TCPC circulation under the control conditions to investigate the basic hemodynamic phenomena associated with the BCPA to TCPC change. The simulated results for the main hemodynamic variables are reported in Table 2, with the corresponding pressure and flow waveforms being illustrated in Figures 3 and 4, respectively. From the results, the BCPA to TCPC change leads to significant hemodynamic changes over the system, such as a marked increase in inferior vena cava (IVC) pressure and a significant decrease in cardiac output and arterial pressure. In addition, there is a slight increase in transpulmonary flow rate upon the BCPA to TCPC change.

The most pronounced changes in flow/pressure waveforms induced by the BCPA to TCPC change were predicted in the IVC. In the BCPA circulation, the IVC blood flow waveform exhibits a typical biphasic shape featured by the presence of two peaks and considerable retrograde flow as has been observed in in vivo studies [34]. After the TCPC operation, only one peak remains and the retrograde flow disappears. At the same time, the pulsatility of the IVC pressure is significantly reduced.

It should be noted that post-TCPC cardiovascular regulation or adaptation has not been considered in the present study. As a consequence, the computed post-TCPC cardiac output and arterial pressure are obviously lower than those reported in previous studies [32].

### 3.2. The Effects of Cardiovascular Properties on the Changes in Pulmonary Flow Rate and Venous Pressure upon the BCPA to TCPC Change

**Figure 5:**
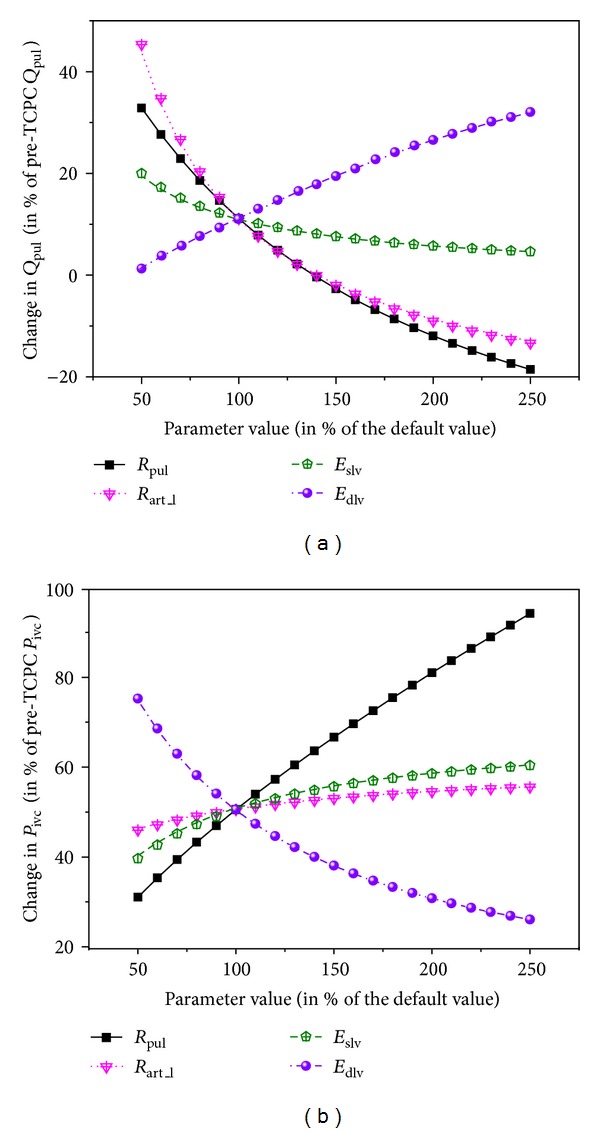
Simulated changes in transpulmonary flow rate (*Q*_pul_ (a)) and venous pressure (*P*_ivc_ (b)) under various cardiovascular conditions upon the change of a BCPA into a TCPC. The results are expressed as percentage changes relative to the pre-TCPC values. *R*_pul_: pulmonary vascular resistance; *R*_art_l_: lower-body arteriolar resistance; *E*_slv_: maximum active elastance of the left ventricle in systole; *E*_dlv_: baseline passive elastance of the left ventricle in diastole.

Figure 5 shows the simulated changes in pulmonary flow rate (a) and IVC pressure (b) when the BCPA is changed into a TCPC under various cardiovascular conditions as described in Section 2.3. Herein, the results are presented in the form of a percentage change in pulmonary flow rate or venous pressure relative to the pre-TCPC value to favor a quantitative comparison among the effects of different parameters. Pulmonary vascular resistance (*R*_pul_) and lower-body arteriolar resistance (*R*_art_l_) are observed to have the most significant influence on the change in transpulmonary flow associated with the TCPC operation. With an extremely high *R*_pul_ or *R*_art_l_, pulmonary flow may even decrease after the TCPC operation. Interestingly, with ventricular diastolic dysfunction (herein characterized by increased ventricular chamber stiffness in diastole), the BCPA to TCPC change is accompanied by a larger increase in pulmonary flow. Relatively, ventricular systolic function only has a mild effect. The change in central venous pressure (herein refering to pressure in the IVC) is determined primarily by pulmonary vascular resistance and ventricular diastolic function, while it is less affected by lower-body arteriolar resistance and ventricular systolic function.

### 3.3. The Effects of Mitral Valve Regurgitation on the Changes in Pulmonary Flow Rate and Venous Pressure upon the BCPA to TCPC Change

**Figure 6:**
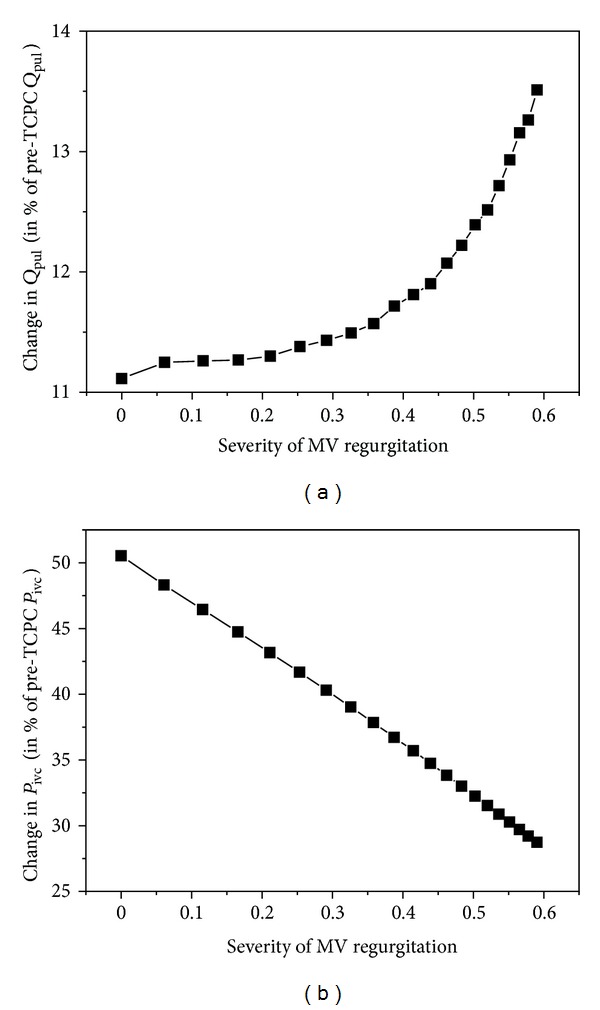
Simulated changes in transpulmonary flow rate (*Q*_pul_ (a)) and venous pressure (*P*_ivc_ (b)) under various mitral valve (MV) regurgitation conditions upon the change of a BCPA into a TCPC. The results are expressed as percentage changes relative to the pre-TCPC values.

Figure 6 shows the changes in pulmonary flow rate (a) and venous pressure (b) plotted as a function of the severity of mitral valve regurgitation (expressed as the ratio of the retrograde to forward transmitral flow rate over a cardiac cycle). From the results, the presence of mitral valve regurgitation tends to improve pulmonary flow, although the degree of improvement is fairly limited (within 3%). Moreover, mitral valve regurgitation attenuates the increase in venous pressure following the BCPA to TCPC change.

## 4. Discussion

Many risk factors associated with poor clinical outcomes in Fontan operation have been identified in clinical studies [6–11], which typically include high pulmonary vascular resistance, low ventricular ejection fraction, increased ventricular diastolic stiffness, and atrioventricular valve regurgitation. These findings have provided valuable information for refining the management of patients in both the preoperative and postoperative stages. However, it remains unclear how these risk factors influence the transient hemodynamic changes upon the change of a BCPA to a TCPC. The issue has been addressed in the present study using a computational method. The major findings of the study include the following: (1) the change in transpulmonary flow upon the BCPA to TCPC change is determined primarily by the pulmonary vascular resistance, the lower-body arteriolar resistance, and the diastolic function of the left ventricle, while it is less affected by the systolic function of the left ventricle and mitral valve regurgitation and (2) the change in venous pressure is dependent strongly on the pulmonary vascular resistance and the diastolic function of the left ventricle.

The importance of a low pulmonary vascular resistance in improving clinical outcomes in post-TCPC patients has been well documented [8, 9]. The present study provides additional evidence to support the fact that the beneficial role of a low pulmonary vascular resistance is also justifiable at the moment of BCPA to TCPC change in terms of improving the transpulmonary flow and restricting an excessive increase in central venous pressure. Compared to pulmonary vascular resistance, systemic vascular resistance has been considered as less important for the regulation of cardiac output in post-TCPC patients [35, 36]. Our study, however, demonstrates that the status of the systemic vascular resistance in a BCPA circulation has a considerable influence on perioperative hemodynamic changes upon the BCPA to TCPC change. For example, in the presence of a low lower-body vascular resistance, a larger perioperative increase in transpulmonary flow can be expected, which partly justifies the use of vasodilator drugs in the treatment of single-ventricle patients.

**Figure 7:**
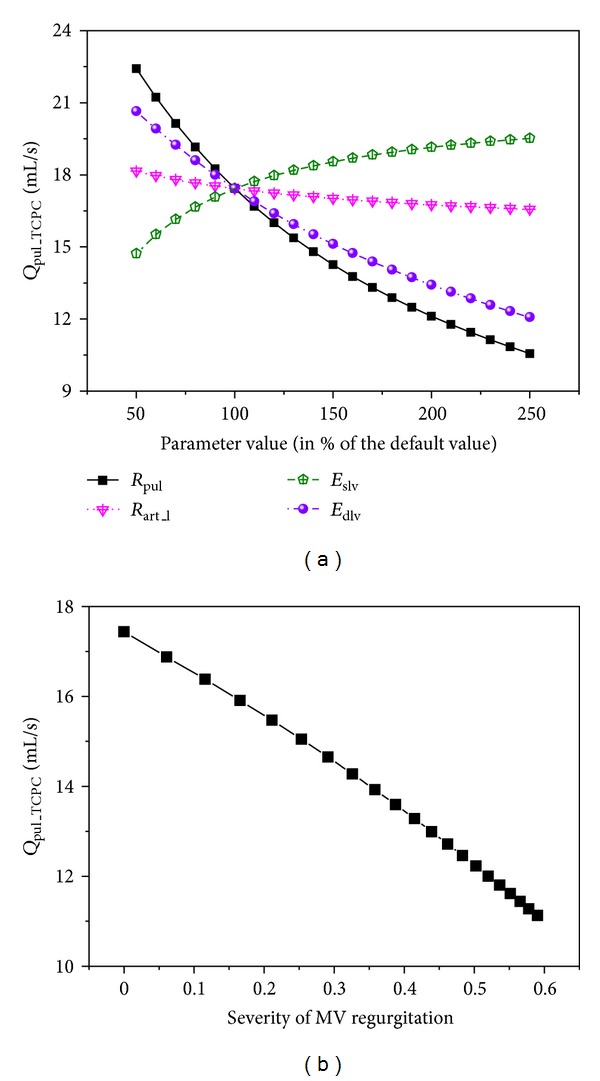
Simulated variations in pulmonary flow rate (*Q*_pul_TCPC_) with cardiovascular properties (a) and the severity of mitral valve regurgitation (b) after the TCPC operation.

It is interesting to find that diastolic dysfunction of the left ventricle (herein characterized by increased chamber stiffness in diastole) and mitral valve regurgitation enhance the increase in pulmonary flow upon the BCPA to TCPC change. This finding implies that the BCPA to TCPC change may not induce a significant reduction in transpulmonary flow in patients with diastolic dysfunction or mitral valve regurgitation. On the other hand, when the regulation of pulmonary flow after the TCPC operation is concerned, both diastolic dysfunction and mitral valve regurgitation are found to have a significantly adverse effect (see Figure 7). These abnormalities hamper a further increase in pulmonary flow via postoperative compensatory adaptations (e.g., reduction in venous compliance or increase in blood volume), thereby leading to poor long-term clinical outcomes as those which have been confirmed in post-TCPC patients [6–11]. As for ventricular systolic function, although it has little effect on perioperative hemodynamic changes in comparison with diastolic function, it plays a considerable role in regulating transpulmonary flow after the TCPC operation, especially under pathological conditions (see Figure 7(a)).

Although the study has provided some new findings to enrich the current clinical understanding, there are limitations that may influence the clinical application of the findings. A significant limitation may stem from building the models based on in vivo data collected from a limited number of patients. Incorporating in vivo data obtained from a larger patient group would allow the models to better capture the typical hemodynamic characteristics in the patient cohort. On the other hand, the cardiovascular properties of any single patient may deviate considerably from the population-averaged trends, implying that a comprehensive consideration of the patient-specific cardiovascular conditions is mandatory for a reasonable patient-specific prediction of the hemodynamic changes associated with a surgery. Moreover, under in vivo conditions, both short-term compensatory regulation and chronic adaptation are likely to occur after the TCPC operation to maintain arterial pressure and restore cardiac output [37], which would further complicate the roles played by different cardiovascular properties in hemodynamic regulation. These mechanisms, however, have not been incorporated in the present study where the BCPA circulation is converted to a TCPC circulation simply by altering the configuration of the vena cava to pulmonary artery connection without changing the values of other model parameters. As we have mentioned previously, the present study focuses on investigating the transient hemodynamic changes upon the BCPA to TCPC change when the effects of cardiovascular regulation or adaptation have not appeared. Therefore, the findings of the present study should not be used to interpret the long-term hemodynamic consequence of the TCPC operation.

## 5. Conclusions

In the present study, computational models were used to quantify the effects of various cardiovascular properties on hemodynamic changes associated with the change of a BCPA into a TCPC in the Fontan operation. Some new insights have been obtained, which include the following: (1) pulmonary vascular resistance and lower-body vascular resistance are the main determinants of perioperative hemodynamic changes; (2) ventricular diastolic dysfunction and mitral valve regurgitation do not deteriorate perioperative hemodynamic changes, although they significantly impair the capability of the cardiovascular system to further improve pulmonary flow after the operation; and (3) ventricular systolic function has little effect on perioperative hemodynamic changes. These findings may serve as a useful theoretical reference for physicians to assess the perioperative risk of the TCPC operation based on preoperative measurement of cardiovascular function.

## Figures and Tables

**Table 1:** Default parameter values used in the BCPA circulation model under resting conditions.

Heart	*E*_ras_ = 0.26	*E*_rad_ = 0.36	*E*_las_ = 0.5	*E*_lad_ = 0.7	*E*_lvs_ = 6.64	*E*_lvd_ = 0.18
	*T*_0_ = 0.67	*T*_vcp_ = 0.246	*T*_vrp_ = 0.147	*S*_ra_ = *P*_ra_∗0.0005	*S*_la_ = *P*_la_∗5*E* − 4	*S*_lv_ = *P*_lv_∗5*E* − 4

Cardiac valves	*L*_mv_ = 1.4*E* − 3	*B*_mv_ = 8*E* − 5	*R*_mv_ = 8*E* − 3	*L*_av_ = 1.4*E* − 3	*B*_av_ = 1.2*E* − 4	*R*_av_ = 1.2*E* − 2

Pulmonary circulation	*C*_pt_ = 0.144	*L*_pt_l_ = 4*E* − 3	*R*_pt_l_ = 1*E* − 3	*L*_pt_r_ = 4*E* − 3	*R*_pt_r_ = 1*E* − 3	
	*L*_pua_l_ = 6.7*E* − 3	*R*_pua_l_ = 0.235	*C*_pua_l_ = 0.217	*L*_puc_l_ = 4*E* − 3	*R*_puc_l_ = 0.173	*C*_puc_l_ = 2.35
	*L*_puv_l_ = 6.7*E* − 3	*R*_puv_l_ = 0.103	*C*_puv_l_ = 0.97	*L*_pua_r_ = 6.7*E* − 3	*R*_pua_r_ = 0.235	*C*_pua_r_ = 0.217
	*L*_puc_r_ = 4*E* − 3	*R*_puc_r_ = 0.173	*C*_puc_r_ = 2.35	*L*_puv_r_ = 6.7*E* − 3	*R*_puv_r_ = 0.103	*C*_puv_r_ = 0.97

ASD	*L*_ASD_ = 5*E* − 4	*R*_ASD_ = 1*E* − 3				

Aorta	*L*_ao_l_ = 3*E* − 2	*R*_ao_l_ = 6.5*E* − 2	*L*_ao_u_ = 1.5*E* − 2	*R*_ao_u_ = 0.15	*C*_ao_ = 0.267	

Vena cava	*L*_ivc_ = 7.5*E* − 3	*R*_ivc_ = 2*E* − 2	*C*_ivc_ = 3.95	*L*_svc_ = 3.8*E* − 3	*R*_svc_ = 6.4*E* − 2	*C*_svc_ = 0.54

Systemic circulation	*L*_art_l_ = 1.5*E* − 2	*R*_art_l_ = 4.22	*C*_art_l_ = 0.13	*L*_cap_l_ = 4.5*E* − 3	*R*_cap_l_ = 0.32	*C*_cap_l_ = 6*E* − 2
	*L*_ven_l_ = 9*E* − 3	*R*_ven_l_ = 6.5*E* − 2	*C*_ven_l_ = 22.0	*L*_art_u_ = 7.5*E* − 3	*R*_art_u_ = 2.91	*C*_art_u_ = 1.8*E* − 2
	*L*_cap_u_ = 2.3*E* − 3	*R*_cap_u_ = 0.75	*C*_cap_u_ = 8.5*E* − 3	*L*_ven_u_ = 4.5*E* − 3	*R*_ven_u_ = 0.15	*C*_ven_u_ = 3.0

Notation of parameters: *E*: elastance; *S*: viscoelastic coefficient; *T*: time; *R*: resistance; *L*: inertance; *C*: compliance; *B*: Bernoulli's resistance. Please refer to Figure 1 for the locations of the parameters in the model. Units of parameters: *E*: mmHg·mL^−1^; *S*: mmHg·s·mL^−1^; *T*: s; *R*: mmHg·s·mL^−1^; *L*: mmHg·s^2^·mL^−1^; *C*: mL·mmHg^−1^; *B*: mmHg·s^2^·mL^−2^.

**Table 2:** Simulated hemodynamic variables for the BCPA circulation and the TCPC circulation under resting conditions compared with the measured data.

	Glenn (mea.)	Glenn (sim.)	TCPC (sim.)
m*P*_svc_ (mmHg)	14.0 ± 2.0 [31]; 10.0 [18]	10.0	8.5
m*P*_ivc_ (mmHg)	5.4 ± 3.2 [32]	5.3	8.0
m*P*_pa_ (mmHg)	12.3 ± 3.1 [33]; 9.0 [18]	9.0	7.8
m*P*_la_ (mmHg)	5.0 [18]	5.0	3.4
m*P*_art_ (mmHg)	72.0 [18]; 73 ± 10 [32]	72.1	45.6
SV (mL)	20.1*	20.1	11.7
EDV (mL)	32.4*	32.5	19.4
EF (%)	62 ± 7 [31]	61.9	60.2
m*Q*_pul_ (mL/s)	15.6 [18]	15.7	17.4
CO (mL/s)	31.2 ± 7.2 [31]; 28.9 [18]	30.0	17.4

Notation of hemodynamic variables: m*P*_svc_, m*P*_ivc_, m*P*_pa_, m*P*_la_, and m*P*_art_ represent the mean pressure in the superior vena cava, the inferior vena cava, the pulmonary artery, the left atrium, and the systemic arteries, respectively; SV, EDV, and EF refer, respectively, to the stroke volume, the end-diastolic volume, and ejection fraction of the left ventricle; m*Q*_pul_ denotes the mean blood flow rate through the pulmonary circulation; and CO is cardiac output. The data marked with “*” were derived from CO and EF by assuming a cardiac duration of 0.67 s.
